# Formulation of the Challenges in Brain-Computer Interfaces as Optimization Problems—A Review

**DOI:** 10.3389/fnins.2020.546656

**Published:** 2021-01-21

**Authors:** Shireen Fathima, Sheela Kiran Kore

**Affiliations:** ^1^Department of Electronics and Communication Engineering, HKBK College of Engineering, Bengaluru, India; ^2^Department of Electronics and Communication Engineering, KLE Dr. M. S. Sheshagiri College of Engineering and Technology, Belgaum, India

**Keywords:** electroencephalogram, brain-computer interface, optimization, evolutionary algorithms, review of EEG

## Abstract

Electroencephalogram (EEG) is one of the common modalities of monitoring the mental activities. Owing to the non-invasive availability of this system, its applicability has seen remarkable developments beyond medical use-cases. One such use case is brain-computer interfaces (BCI). Such systems require the usage of high resolution-based multi-channel EEG devices so that the data collection spans multiple locations of the brain like the occipital, frontal, temporal, and so on. This results in huge data (with high sampling rates) and with multiple EEG channels with inherent artifacts. Several challenges exist in analyzing data of this nature, for instance, selecting the optimal number of EEG channels or deciding what best features to rely on for achieving better performance. The selection of these variables is complicated and requires a lot of domain knowledge and non-invasive EEG monitoring, which is not feasible always. Hence, optimization serves to be an easy to access tool in deriving such parameters. Considerable efforts in formulating these issues as an optimization problem have been laid. As a result, various multi-objective and constrained optimization functions have been developed in BCI that has achieved reliable outcomes in device control like neuro-prosthetic arms, application control, gaming, and so on. This paper makes an attempt to study the usage of optimization techniques in formulating the issues in BCI. The outcomes, challenges, and major observations of these approaches are discussed in detail.

## 1. Introduction

Brain computer interfaces (BCI) are an important application of electrocephalogram (EEG) signals (Navalyal and Gavas, 2014). The usage of EEG signals in such an application other than medical use cases is due to the availability of affordable EEG devices. Also, the effectiveness of the algorithms used in the conventional BCI pipelines play a major role in this regard. In general, BCI system's pipeline consists of the following blocks: pre-processing of the EEG data, event-related potential (ERP) analysis, extraction of features, and classification of data (Sinha et al., 2015b), and so on. The effectiveness of these blocks can be measured as a function of time complexity, computational resources required, and the accuracy of the algorithms. With respect to enhancing the accuracy of the algorithms, various attempts have been laid in making them robust by finding optimal tuning parameters for them. This is however, not a straight forward task as designing of effective objective functions and the choice of optimization problems is a very challenging task. Hence, there is a rich source of EEG and BCI literature that mainly focuses on using optimization techniques and their enhanced variants in the BCI pipelines. This paper aims at studying the usage of optimization from the view point of the application in BCI, i.e., with respect to the standard BCI pipelines.

Optimization schemes play a major role in most of the engineering problems where direct understanding of the system is not feasible. In case of EEG analysis, it is difficult to ascertain the exact locations of the neuronal firings owing to volume conduction. Invasive EEG can aid in this regard but cannot be applied in day-to-day scenarios for all the participants. In such cases, the domain knowledge can be of great help but in the lack of this knowledge for novel BCI systems, arriving at proper tuning parameters of BCI is very difficult. The system needs to be tested over a large set of parameters available by repeating the experiments for multiple times, which again is not a practical solution. This has motivated the BCI community to adopt optimization schemes in their pipelines.

The usage of optimization techniques in BCI applications requires the proper understanding of the objectives and the domain knowledge plays a vital role here. For instance, in the EEG channel selection problem, the domain knowledge would make the analyst to select the channels which are relevant to the task type. However, it can be seen that optimization tools would recommend some other channels but would enhance the accuracy of the BCI much more than what the domain knowledge-based channel selection might have done. But this set of channels might not be consistent across participants. Hence, it is necessary to have well-defined objective functions while using the optimization algorithms. This study summarizes the BCI applications that have used optimization and also the parameters of BCI are reviewed in detail. This would aid the reader in appreciating the essence of optimization in BCI-based applications.

The rest of the paper is organized as follows. Section 2 of the paper reviews the existing literature that uses optimization in various BCI pipelines. Section 3 discusses the challenges involved in adopting optimization schemes in BCI. Section 4 summarizes the paper and also the possible medical use cases of optimization in EEG analysis. The paper concludes in section 5 with pointers to the guidelines in using optimization techniques in BCI.

## 2. Formulation of Optimization Problems in BCI

Optimization is a technique that is performed by comparing different solutions to find an optimal solution. Such algorithms aim to maximize or minimize an error function (usually termed as an objective function). The objective function is a representative of the model's tuning parameters. Optimization has seen tremendous applications in various branches of science and engineering. Optimization techniques helps to arrive at optimal parameters in the lack of domain knowledge or when it is not feasible to test the system directly. For instance, in case of EEG feature selection for a novel stimulus, the physical interpretations of most of the non-linear, time/frequency features is not possible with respect to the task.

The underlying mechanisms of converging toward an optimal solution in case of optimization is very well correlated to various naturally occurring phenomena. Hence, over the past few decades, researches have been motivated from nature in designing such algorithms. Such algorithms are termed as evolutionary algorithms which is a form of stochastic optimization. Most widely used evolutionary algorithms are Particles Swarm Optimization (PSO), Genetic Algorithm (GA), Differential evolution (DE), Ant Colony Optimization (ACO), Artificial Bee Colony (ABC), and so on. We noticed that most of the BCI-based applications have made use of evolutionary algorithms in deriving the optimal tuning parameters for various BCI pipelines.

The following section reviews the formulation of optimization problems around building efficient BCI pipelines. It is to be noted that during this review, we came across various datasets like motor imagery (MI), emotion recognition, visual evoked potential (VEP), sleep apnea detection, mental, or cognitive tasks, ERP analysis, and so on. We also found that the task of EEG classification is mostly carried out using standard machine learning classifiers (having inbuilt optimization mechanisms) and hence, the explicit usage of optimization (by the researchers) is missing in these cases. Hence, we have excluded the EEG classification block in this review.

### 2.1. Optimization of EEG Pre-processing

Noisy signals occurring due to multiple factors during EEG data collection contaminates the signal. The noises inherent in EEG can be classified as follows (Zhang et al., 2016):

(i) Technical artifacts

1. Electrode related artifacts: The noise related to electrodes can be due to improper placement, electrode slippage, varying impedance, poor condition of the sensors, and so on. Usually the wet electrodes, if not cleaned properly, gets rusted, and deteriorates the signal.
2. Sweating: The sweating on the scalp can vary the impedance of the electrodes and lead to unwanted artifacts in the signal.
3. Power line interference: strong signals resulting from A/C supplies contaminates the signal which basically adds a sharp peak at around 50/60 Hz.

(ii) Physiological Artifacts

1. Electrooculargram (EOG) artifacts: These are mainly caused due to eye blinks or eye movements which adds up as a high amplitude signal upon the EEG signal. This artifact mainly affects the frontal channels due to their vicinity from the eyes (Sinha et al., 2015a). Most of these artifacts falls below 4–5 Hz range (Gavas et al., 2020).
2. Electrocardiogram (ECG) artifacts: This mostly occur on the electrodes placed near to the blood vessels, thereby resulting in an unwanted signal centered around 1.2 Hz due to the contraction and the expansion of the vessels.
3. Electromyographic (EMG) artifacts: These artifacts are a result of various muscle movements from face and neck and get accumulated on all the EEG channels. The frequency bandwidth of these signals is very large and mostly falls in the frequencies above 30 Hz.

The process of removing these noises from EEG is referred to as the pre-processing stage. Various studies to remove these noise exists, however, the number of studies using optimization schemes in this process is limited. This can be attributed to the nature of solving the EEG pre-processing problem. We pick some of the studies which have used optimization algorithms in this direction and the summary is presented in Table 1. The table summarizes the task type i.e., the type of artifact removal and the optimization algorithm used for that task.

**Table 1:** Summary of optimization schemes in EEG artifact removal studies.

**References**	**Task**	**EEG data**	**Optimization** **algorithm**
Ahirwal et al. (2012)	Adaptive filtering	Simulated EEG and real VEP	PSO
Ahirwal et al. (2013)	Adaptive noise cancellation	Simulated VEP, real VEP, real sensorimotor evoked potential	PSO, ABC and Cuckoo search
Ahirwal et al. (2014)	Adaptive noise cancellation	MI	Bounded Range ABC
Priyadharsini and Rajan (2014)	ECG component removal from EEG	Simulated data	Variants of memetic algorithm and GA
Wang et al. (2010)	Trial pruning by removing artifacts	MI	GA
Gupta and Palaniappan (2011)	Eye blink artifact removal	BCI MI	Variant of GA
Alyasseri et al. (2017)	Power line and EMG noise removal	Various mental tasks	Hybrid β-Hill climbing
Suja Priyadharsini et al. (2016)	EOG and ECG artifacts removal	Simulated data	Artificial immune system algorithm
Pereira et al. (2016)	EOG and EMG artifacts removal	Simulated data	Variant of GA
Quazi and Kahalekar (2017)	EMG, EOG and ECG artifacts removal	EEG added with sleep apnea ECG and EOG	Firefly + Levenberg Marquardt algorithm

The objective functions involved in optimization based EEG noise cleaning can be any of the following:

- *Minimizing the error between the desired and actual EEG* (Pereira et al., 2016).
- *For obtaining optimal tuning parameter weights for the filtering algorithms used*. These weights in turn are derived using the objective of minimizing the error as discussed above. For instance, Alyasseri et al. (2017) used optimization to obtain optimal wavelet parameters for signal denoising. The studies in Priyadharsini and Rajan (2014) and Suja Priyadharsini et al. (2016) showed the usage of optimization algorithms to enhance the capabilities of adaptive network-based fuzzy inference systems in denoising the EEG signals. Similarly, the authors in Quazi and Kahalekar (2017) used Firefly + Levenberg Marquardt optimization algorithms for tuning the neural networks to adaptively filter the artifacts from EEG.
- *Minimizing the mutual information (MI) between the actual EEG and the corrupted EEG*. The works of Gupta and Palaniappan (2011) showed the reduction in power spectral density of eye blink artifacts using genetic algorithms to minimize the MI between the corrupted and the desired EEG signal.

### 2.2. Optimization of ERP Extraction

Event related potential detection in EEG is an important part in the analysis of various mental activities. ERP is a special case of EEG analysis which is indicative of the direct effects of motor, sensory, or cognitive functions. The estimation of ERP is done by averaging the measurements over an ensemble of trials. This approach requires many trials in order to suppress the underlying noise in EEG. Filtering can solve the issue of noise removal to some extent but the filter parameters needs to be tuned based on the statistical properties of the signal. If the parameters are not tuned properly, it may then result in suppressing the ERPs in the EEG. Hence, optimization plays a very important role in this case. Adaptive filtering serves to be beneficial in this regard as noise cancelers (Ahirwal et al., 2012, 2013, 2014). The authors in Ahirwal et al. (2014) show that through ABC optimization, the performance of adaptive filtering can be enhanced as compared to the conventional LMS and RLS filtering. The objective function defined in Ahirwal et al. (2014) is the minimization of the mean squared error by selecting optimal weights in the adaptive filter.

### 2.3. Optimizing the Problem of Feature Selection

Feature vectors usually comprise of high dimensions and this makes the feature selection an important tool for the classification problems. The idea of feature selection can be categorized into three types (Liu et al., 2010), namely,

- *Filter method*: deals with selection of subset of features by analysing the data characteristics without involving the learning algorithm in the process. As a result, the advantage of these methods is that they do not have any bias toward the learning models. Examples of filter methods are Relief, Correlation-based Feature Selection, Consistency, C4.5, minimum redundancy–maximum relevance (mRmR) (Ramos et al., 2016) and so on.
- *Wrapper method*: selects the subset of features based on the performance of the features on the learning algorithm during the evaluation step. Examples involve using optimization techniques like GA with the objective of maximizing the cross validation accuracy (Bhattacharyya et al., 2014; Pal et al., 2014; Xu et al., 2014; Ramos et al., 2016; Baig et al., 2017; Liu et al., 2017; Ramos and Vellasco, 2018; Ghosh et al., 2019), classification error (Wang and Veluvolu, 2017), unsupervised classification (Kimovski et al., 2015), similarity score and clustering validity index (Bhattacharyya et al., 2013; Rakshit et al., 2013), or classifier parsimony Cîmpanu et al. (2017).
- *Embedded method*: feature selection is incorporated as a part of the model's training process. The relevance of the features is found by evaluating their utility for optimizing the learning algorithm's objective function. The authors in Yin et al. (2017) used the maximization of geometric distance (margin between the targets) in the learning algorithm.

The design of filter methods is simple, i.e., they are either based on forward selection or backward elimination and feature testing criterion which is based on a certain criterion. Hence, they are easy to understand and to implement and thus they are fast in execution. Since, the wrapper and embedded methods are linked to the learning process, their accuracy is higher in comparison to the filter method. Embedded methods are basically a fusion of filter and wrapper methods. Wrappers typically use cross-validation kind of mechanisms for accuracy computation that prevents overfitting. This makes them slower and leads to lack of generality. However, most of the works are found to use the wrapper approach as it is easier to formulate the objective function as a wrapper when compared to a filter and also the accuracy provided by wrappers are higher. The works of Ramos et al. (2016) showed that wrapper methods are better over filters. These feature selection algorithms either return a subset of features or the weights that signify the relevance of the features. Hence, based on the output, the feature selection algorithms can be classified into subset selection or feature weighting.

The feature extraction stage of EEG analysis deals with extracting frequency and time domain features which can be used as the compact representation of the EEG data. This is then fed as an input to various machine learning-based classification blocks. The features extracted have high dimensionality (Kimovski et al., 2015) that can increase the processing time and can result in the inclusion of outliers as features because of poor signal-to-noise ratio of EEG (Tacchino et al., 2020). These factors culminates in reduced accuracy of the BCI system. Hence, selection of appropriate subset of features is a vital step in the analysis of EEG data. In this stage, the features with enhanced discriminative power are used to carry out the further steps. It is to be noted that most of the times, the conventional feature selection algorithms aim to select features with high variances. This at times does not improve the overall accuracy of the system. The major reason could be the presence of redundant features. However, this problem is not a straight-forward task to solve. Many standard feature selection tools are available (Giorgio, 2020) to solve these issues. In the interest of the current paper's scope, the ones using optimization techniques in case of EEG are summarized in Table 2.

**Table 2:** Summary of optimization in EEG-based feature selection studies.

**References**	**Algorithm**	**Task**	**Accuracy**	**Number of** **classes**
Rakshit et al. (2013)	Artificial bee colony	MI	64.29	2
Kimovski et al. (2015)	Parallel multi-objective optimization	MI	100	2, 3
Xu et al. (2014)	Particle swarm optimization	MI	78	2
Bhattacharyya et al. (2014)	DE	MI	99.41, 87.99	2
Pal et al. (2014)	Bacterial foraging algorithm	MI	80.29	2
Bhattacharyya et al. (2013)	DE variant	MI	94	3
Yin et al. (2017)	Transfer recursive feature elimination	Emotion classification	75+	2
Cîmpanu et al. (2017)	Single and multi-objective Genetic algorithm	Memory load detection	<14% (Error rate)	2
Liu et al. (2017)	Firefly algorithm and learning automata	MI	70.2	4
Eslahi et al. (2019)	Genetic algorithm	MI	84 (max)	4
Fernandez-Fraga et al. (2018)	Ant colony optimization	SSVEP BCI	82.76	–
Wang and Veluvolu (2017)	Evolutionary algorithm	MI	83	4
Ramos et al. (2016)	Genetic algorithm	MI	93.71	2
Baig et al. (2017)	Differential evolution	MI	95	3
Ghosh et al. (2019)	Grey wolf optimization	Silent speech classification	65	5
Ramos and Vellasco (2018)	Quantum- inspired evolutionary algorithm	MI	96.86	2
Selim et al. (2018)	hybrid bio-inspired algorithms	MI	78.55, 86.6,85	4,3,4

### 2.4. Optimization of EEG Channel Selection

For any EEG-based application, the selection of channels that is physiologically significant to the system in hand, is of paramount importance. The EEG data acquired is multichannel in nature. It is advisable to work on a subset of the channels instead of considering the whole. This is because, setting up the EEG system on a participant with many channels is cumbersome and time consuming. It also leads to the inconvenience of the participant which might reflect in lack of attention or distraction during the actual data collection. Apart from these subject-specific issues, this also adds to the increased computational complexity of the overall EEG application. Channel reduction is of great interest in designing portable EEG devices for detecting the onset of epileptic seizures hours before they prevail in order to provide early interventions. Such portable systems would need algorithms which are fast and the hardware smaller in size. This makes the usage of channel selection a important research problem in the EEG community. The main objectives of EEG channel selection are: (i) Reduction in dimensionality and providing faster processing, (ii) improving the performance of the model created, and (iii) identification and localization of the brain regions that are responsible for the given activity. Many efforts have been laid toward this direction of achieving an optimal subset of channels. It was realized in the EEG research community that these optimal channel sets can be achieved more easily using optimization tools and this benefited more than considering the EEG channels that are known to be responsible for the task. For instance, the brain region corresponding to motor functions is located in the central region. Hence, it is more appealing to consider the central EEG channels for motor imagery-based analysis. However, due to volume conduction, the locations in the vicinity of central channels would also carry some information regarding the motor imagery. The overlap in information among these channels depends on several factors like the subjective nature of the skull shape, the type, and the sensitivity of the EEG used, and so on. Hence, instead of directly selecting the central channels for motor tasks, the selection of channels has to be personalized which can be done using optimization tools. Table 3 surveys some of the most relevant works in this regard. The accuracy obtained for each of these approaches are also provided. Since, each of these studies used different EEG devices/datasets and subjects, we also report the improvement in accuracy over the state-of-the-art techniques (provided in brackets).

**Table 3:** Summary of optimization in EEG-based channel selection studies.

**References**	**Algorithm**	**Task**	**Accuracy** **(improvement)**	**Number** **of classes**	**Data** **reduction**
Jin et al. (2008)	Discreet particle swarm	Directional moving	77.54 (7.38)	4	Yes
Hasan and Gan (2009)	Multi-objective PSO	MI	57 (NA)	3	Yes
Hasan et al. (2010)	Multi-objective evolutionary algorithm based on decomposition	MI	58 (NA)	3	Yes
Handiru and Prasad (2016)	Iterative multi-objective optimization	MI	80 (7)	2	No
Yang et al. (2012)	Genetic neural mathematic method	MI	80, 86, 82 (NA)	2	Yes
Yang et al. (2013)	Time-spatial optimization	MI	78 (NA)	2	No
Shan et al. (2015)	A novel algorithm based on Relief	MI	85.2 (31.7), 94.1 (8), 83.2 (19.7)	2 and 4	Yes
Arican and Polat (2020)	Binary particle swarm optimization	Speller systems	90, 89.8(NA)	4	Yes
Lv and Liu (2008)	Common spatial pattern + Particle swarm optimization	MI	83,92(NA)	2	Yes
Kim et al. (2013)	Binary particle swarm optimization and GA	MI	78 (mean) and 67 (mean)	2	Yes
Kee et al. (2015)	Multi-objective genetic algorithm	P300 and MI	85+ (5.25–8.60)	2	Yes
He et al. (2013)	Rayleigh coefficient maximization based genetic algorithm	MI	80+(NA)	2	Yes
Joseph and Govindaraju (2019)	Glow swarm optimization	MI	92.59 (6.31, 5.48)	2	No
Zhang and Wei (2019)	PSO	MI	91.94	2	Yes
Ghaemi et al. (2017)	Improved binary gravitation search	MI	76.24 (mean) 80 (max)	4	Yes
Shenoy and Vinod (2014)	Iterative optimization technique	MI	90.77 and 81.21	3 and 4	Yes
Arvaneh et al. (2011)	Sparse common spatial pattern	MI	80+(10)	2 and 2	No
Gonzalez et al. (2014)	Multi-objective hybrid real-binary particle swarm optimization	Auditory ERP	95 (6)	2	Yes
Jin et al. (2019)	Regularized common spatial pattern	MI	81.6 (25.2) 87.4 (10.9) 91.9 (6.8)	2,3,2	Yes

The optimal solution to EEG channel selection refers to a subset of channels that has highest relevance for the given stimulus/experiment. Innovative ways of looking at this problem can be formulated as a multi-objective function as follows,

- *Number of channels*: an obvious expectation is to have the minimum number of selected channels.
- *Region of interest (ROI)-based*: obtaining the candidate channels in the vicinity of the regions in brain that are known to produce the neurophysiological activations
- *Classification accuracy-based*: searching for channels that contributes in obtaining high accuracy of task classification. This can also be related to the case of having minimum error rate for the test set data.

It is important to note that for channel reduction/selection problems, the reduction of raw data plays a vital role in reducing the time and space consumption of the system. Downsampling allows the reduction of computational cost while retaining the vital information in the time-series data. As most relevant EEG activity lies in the range of 0.1–50 Hz, downsampling the signal from higher frequencies to 100 Hz is usually carried out in most of the studies like (Hasan and Gan, 2009; Hasan et al., 2010; He et al., 2013; Gonzalez et al., 2014; Shenoy and Vinod, 2014; Kee et al., 2015; Shan et al., 2015; Zhang and Wei, 2019; Arican and Polat, 2020). Though downsampling seems to be a straightforward approach, some studies reduced the data size by first extracting the features (as features are a compact way of looking at the data) and then the features were subjected to principal component analysis (PCA) to further reduce the dimension. The studies mentioned in Table 3 that used this approach are Ghaemi et al. (2017), Hasan and Gan (2009), Jin et al. (2008), and Kim et al. (2013). Few other studies like the ones in Hasan and Gan (2009); Hasan et al. (2010), used both the techniques to reduce the data size. The works by Yang et al. (2012) used time and frequency based feature analysis to reduce the dimension of the data.

### 2.5. EEG Mode Decomposition and Optimization

Mode decomposition of time series signals refers to decomposing a given signal into several realizations which differs in terms of morphological characteristics like frequency response from each other. The summation of all these realizations reproduces the original signal. The realizations are termed as intrinsic mode functions (IMFs). EEG signal mode decomposition becomes important to reconstruct or separate out various neuronal activities (Soler et al., 2020), source localization (Khosropanah et al., 2018), artifact removal (Wang et al., 2015), detection of seizures (Bajaj and Pachori, 2011), and so on.

Various studies have used signal decomposition algorithms like empirical mode decomposition (EMD), ensemble EMD (EEMD), variational mode decomposition (VMD), and so on to decompose physiological signals. Out of these, the VMD algorithm is based on solving an optimization function which in turn makes it robust against the existing mode decomposition algorithms (Gavas and et al., 2018). VMD basically looks at the problem of signal decomposition as an optimization problem by decomposing a 1-dimensional time series into *K* number of modes *u*_*k*_(*t*) as, $x\left(t\right)=\overset{K}{\underset{k=1}{\sum }}u_{k}\left(t\right)$, with the criterion that the signal gets reconstructed ideally fully by summing up the *K* number of modes while the sum of bandwidths of all modes is kept minimum (Dragomiretskiy and Zosso, 2013). Every mode is compact along the mean frequency *w*_*k*_. The method solves a constrained variational function to find optimal *w*_*k*_ and *u*_*k*_ given by,

(1) $$\begin{array}{l} min\left\{\|\underset{k}{\sum }\partial_{t}\left[\begin{array}{c} \left(\delta \left(t\right)+\frac{j}{\pi t}\right)*u_{k}\left(t\right) \end{array}\right]e^{-j\omega_{k}t}{\|}_{2}^{2}\right\}\\ subject\;to\underset{k}{\sum }u_{k}=x \end{array}$$

The reader is requested to get the detailed explanation of the VMD algorithm from Dragomiretskiy and Zosso (2013). The number of IMFs extracted from the decomposing algorithms is mainly application dependent and is often restricted to a certain number by empirical analysis of the central frequencies of the IMFs. We summarize few of the applications wherein VMD or its variants were used (Table 4). Note the number of IMFs extracted in each of the case is different.

**Table 4:** Summary of papers using VMD in EEG signal analysis.

**References**	**Task**	**No. of EEG channels**	**VMD type**	**No. of IMFs**
Rehman and Aftab (2019)	Separation of alpha rhythms	4	Multivariate VMD	5
Gavas et al. (2020)	Blink artifact removal	4 and 57	Multivariate VMD	10
Zhang et al. (2017)	Feature extraction for Seizure detection	Single	Univariate VMD	15
Taran and Bajaj (2018)	Identification of focal EEG	Single	Clustering-VMD (univariate)	2
Bhattacharjee et al. (2018)	Sleep Apnea detection	Single	Univariate VMD	5
Dora and Biswal (2020a)	ECG artifact correction from EEG	Single	modified VMD (univariate)	12
Taran and Bajaj (2019)	Emotion recognition	10 out of 24 used	Univariate VMD	–
Dora and Biswal (2020b)	Ocular artifact suppression	5	Univariate VMD	12
Saini et al. (2019)	Ocular artifact removal	Single	Extended Univariate VMD	2 and 3
Saini et al. (2020)	Muscle artifact supression	Single	Univariate VMD	2
Yücelbaş et al. (2018)	Detection of K-complexes	2	Univariate VMD	–

## 3. Challenges Involved in Optimization of BCI Pipelines

**Figure 1:**
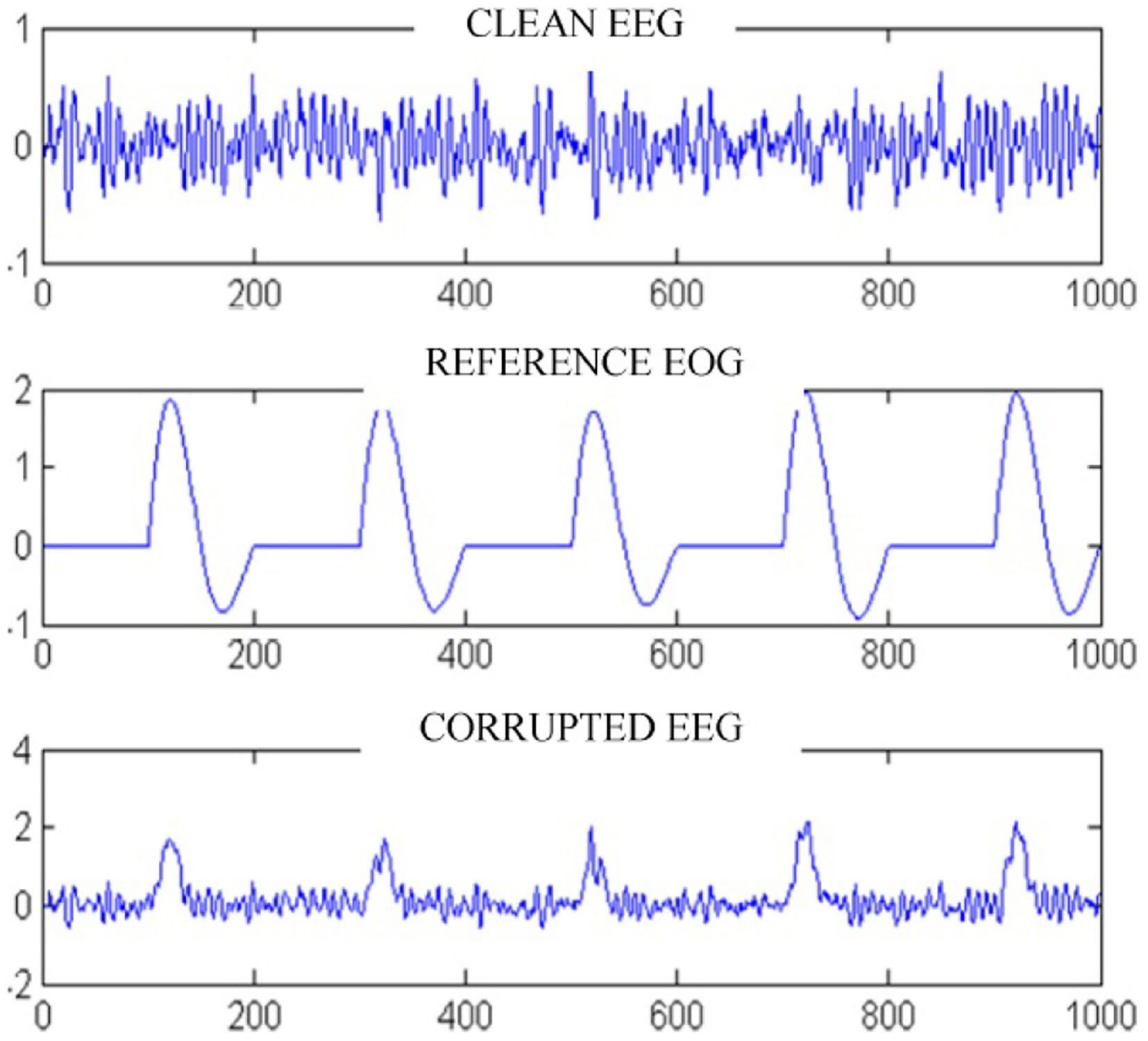
Sample embedding of EEG signal with EOG artifact (adapted from Pereira et al., 2016).

The main issue faced in any EEG-based artifact removal studies, particularly when it comes to the removal of other physiological effects like ECG, EOG from EEG is the absence of exact ground truth (Gavas et al., 2020). Usage of simulated data becomes a straightforward approach of validating the designed noise removal algorithms in such cases. Figure 1 shows a typical approach of generating an EEG signal with an EOG artifact (Pereira et al., 2016). The simulated data can provide the exact start and stop events of the physiological artifact like blink and also the exact morphology of the artifact embedded onto the raw signal. The test cases involving the simulated data performs better with the designed algorithms but the results degrade when it comes to real data. In such cases, the usage of conventional signal processing tools or even optimization-based data driven methods perform somewhat similar, as setting up the proper basis functions is difficult in such cases. However, mode decomposition algorithms are seen to be a better alternatives in such cases (Gavas et al., 2020) involving simulated or real EEG data.

Owing to the higher sampling rates and the increased number of channels in EEG, the amount of processing time and resources required for the EEG data is huge. For instance, decomposing a multi-channel EEG data with a high sampling rate using the MVMD (Rehman and Aftab, 2019) can be very slow, computationally very complex and requires huge amount of memory.

**Figure 2:**
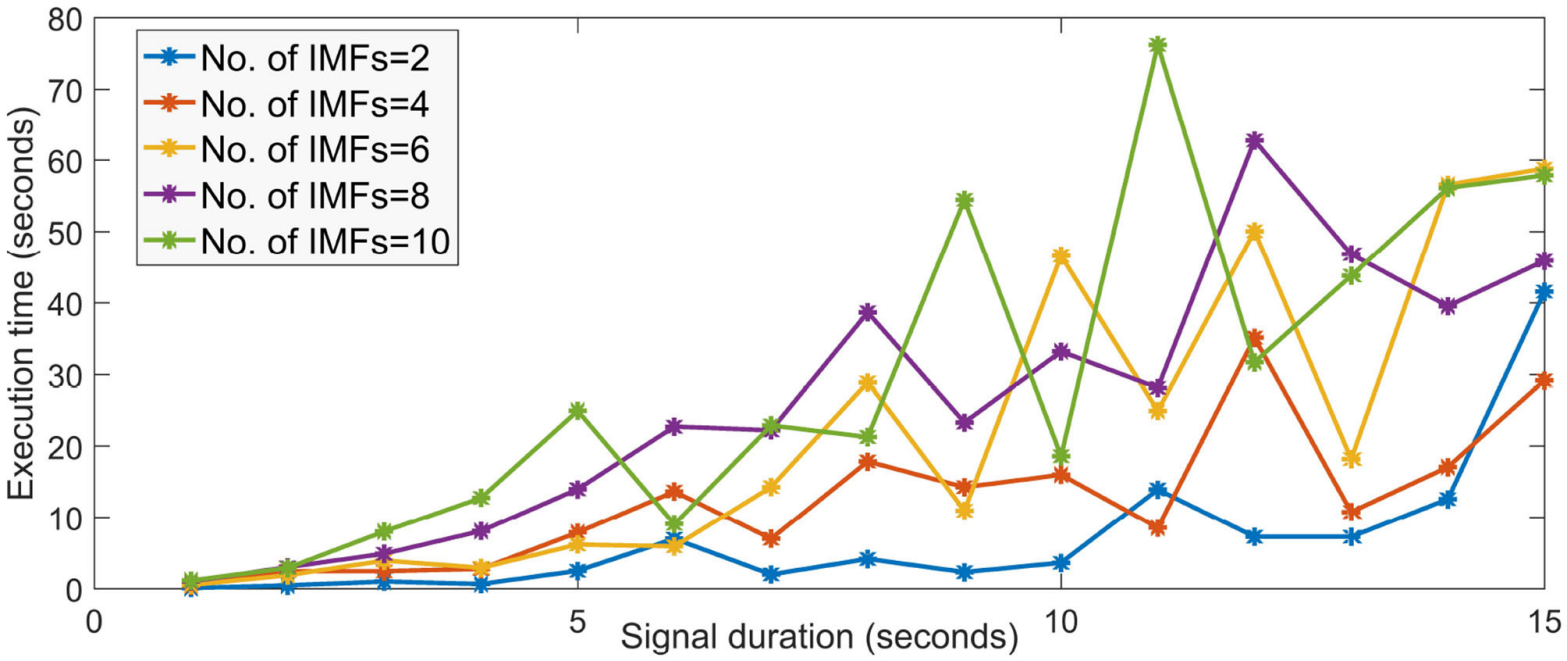
Execution time of MVMD algorithm for different signal duration and different number of IMFs.

To visualize, this, we ran the MATLAB implementation of the MVMD algorithm on a 4 GB RAM, core i5 processor machine by simulating a 4-channel EEG data of various small duration. The execution time is as seen in Figure 2. It is to be noted that the execution time increases drastically as the signal duration and the number of IMFs increases. The number of channels also plays a major role in determining the run time of the algorithm. For higher number of channels and signal duration, the required system memory and time is very large and cannot run on low configuration devices. Same is the case when dealing with such data using evolutionary algorithms which require atleast a good number of iterations (usually more than 100) to converge to a good solution. Also, the fear of converging lately or getting stuck in local minima can always be a major set back in using such optimization schemes in real time BCI.

EEG signal mode decomposition is seen to be beneficial for various applications in BCI. However, as seen in Table 4, the number of IMFs extracted is not constant across studies even for the same BCI task. This inconsistency is commonly addressed as arising due to the nature of the application but the actual fact lies in the nature or the stochasticity of the EEG signal. If EEG signals were deterministic, then the frequency components across the same IMFs across different EEG data would be similar. This would have helped building new applications that make use of mode decomposition without investing much efforts on experimenting on the optimal number of IMF generation.

Consider the problem of EEG channel selection for MI tasks. By domain knowledge it is known that the central channels like C2, CZ, and C3 are well-suited for motor imagery related activities. However, due to the effect of volume conduction, the idea of relying on only the central channels is questionable. Owing to the subjective aspects like the skull size and the nature of EEG sensor, the channels picking up the motor imagery data faithfully, might vary from person to person. In such cases, the usage of personalized channel selection using optimization schemes seems to be an attractive idea (Shireen Fathima, 2019). The major challenge foreseen in this case is the design of the objective function to select the optimal channels. Even if this problem is tackled, the next major issue lies in the selection of optimization algorithm and also initializing the tuning parameters of the algorithm. Researchers have mainly used meta-heuristic algorithms in such cases. As EEG signals are highly stochastic and non-linear in nature, different optimization algorithms can lead to the selection of different EEG channels, for the same participant and for the same task.

**Figure 3:**
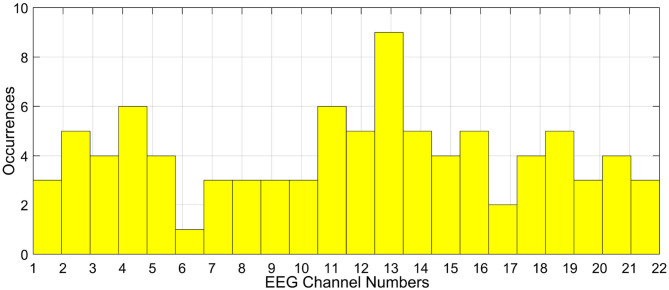
Histogram of selected EEG channels for a 22-channel MI task across participants.

Even the consistency of channel selection across participants for a given optimization algorithm is not possible. For instance, we used the channel selection method (Khushaba et al., 2011) on a motor imagery BCI as mentioned in Shireen Fathima (2019) on a 22-channel EEG data. The resulting histogram of the selected channels across all the participants for the same task is given in Figure 3. The histogram is generated by considering the optimal channel ids for all the participants taken together. It is to be noted that in the figure, the channels are not consistent across all the participants and the generalization of channels is not possible. If same channels were selected as optimal channels, then the histogram would have centered over a small subset of channels. On similar grounds, the results change drastically when different optimization schemes are used for the said purpose. This can really make the task of arriving at a subset of generalized optimal channels to be used during real time BCI challenging, as no algorithm till date yields the same set of optimal channels for the same task and for the same participant.

**Figure 4:**
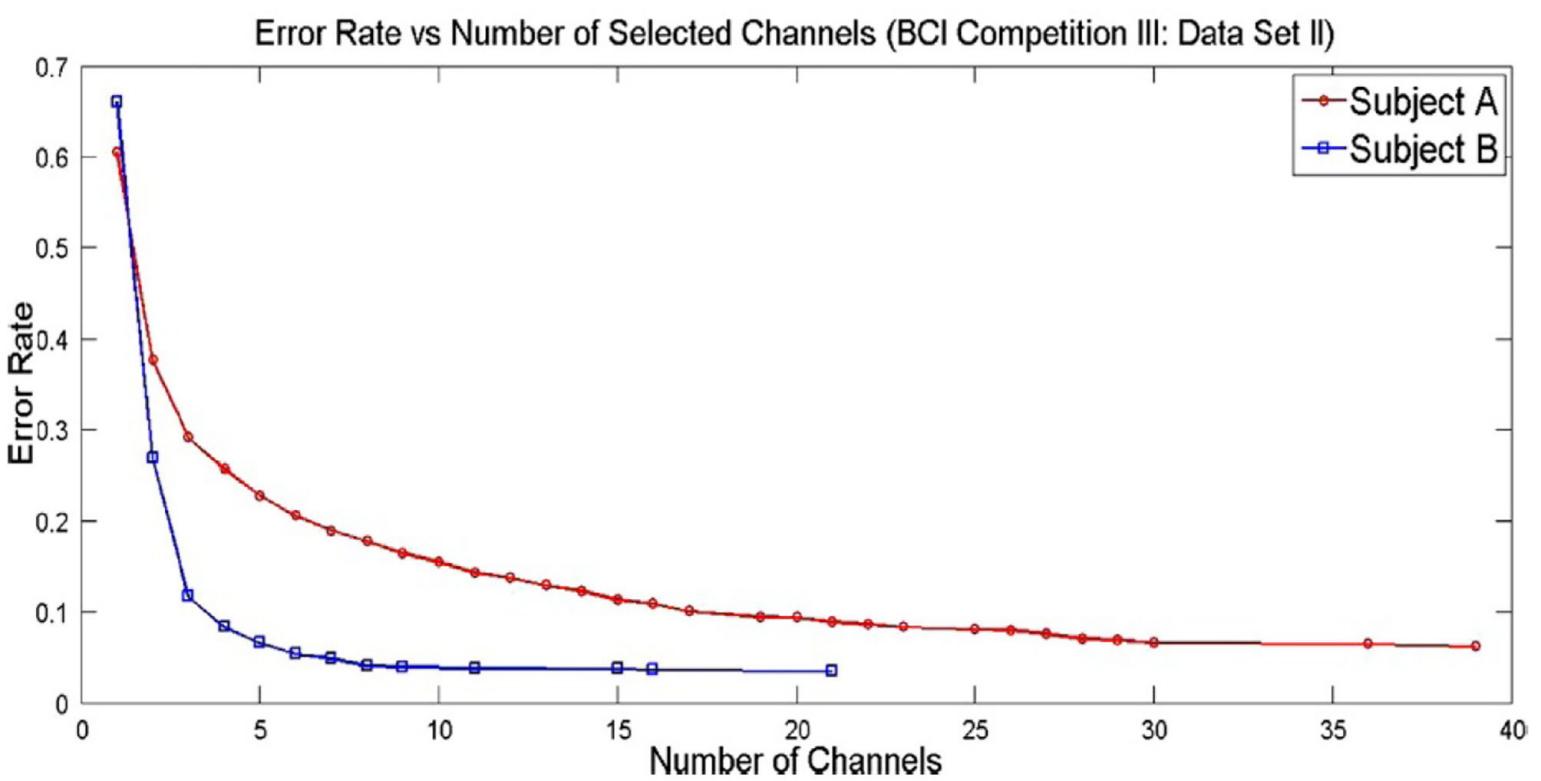
Pareto optimal solutions for a channel selection problem in MI task (adapted from Kee et al., 2015).

Solving multi-objective functions of channel selection like least number of channels and least error rate leads to pareto solutions (as shown in Figure 4) and selecting a pareto optimal solution depends on the researcher or on the application. Figure 4 shows the pareto solutions of error rates at the expense of number of channels (Kee et al., 2015). As the number of channels increase, there is a decrease in error rate. In such cases, it is tricky to settle down to a certain count of channels with a satisfactorily lower error rate.

## 4. Discussions

Usage of optimization has recently gained wide popularity in EEG analysis, mainly in the field of feature selection and channel selection. This can be attributed to the fact that these two tasks are straightforward, majorly relying on the objective of maximizing accuracy of classification tasks. Though channel selection and feature extraction are means of selecting a subset of the data, however, they both vary considerably in nature. Channel selection deals with selecting a subset of optimal channels whereas, feature selection deals with selection of a subset of optimal features. A common practice is to apply feature selection on the subset of optimal selected channels. The selected optimal channels can give insights on the source location of the task being performed. However, the selected features can help understand the signal specific characteristics of the underlying effect. Another common practice that we observed in this field is the usage of evolutionary algorithms. Generally, when non-linear optimization schemes are deployed for EEG based problems, the objective function yields multiple local solutions in cases involving high dimensional search space and for lower values of signal-to-noise ratios. This has attracted the researchers to use meta-heuristic algorithms which work very well for such scenarios. Hence, it is obvious to find a rich source of EEG optimization literature involving meta-heuristic algorithms which is also evident in this review.

Selection of proper objective functions is crucial to any optimization-based problem solving. In case of EEG, this becomes more challenging owing to the non-stationary nature of the signal but at the same time, it comes with added advantages. Table 5 summarizes the objective functions, its advantages and disadvantages in different EEG pipelines. It is evident that optimization when used in any given EEG pipeline comes with its own pros and cons. However, their widespread usage in current times shows the benefits that it has over their conventional counterparts.

**Table 5:** Summary of optimization strategies employed in EEG analysis.

**Task**	**Objective functions**	**Advantages**	**Disadvantages**
Noise cleaning	(1) Minimize error	Output signal resembles desired signal	Knowledge of target signal characteristics is a must
	(2) Obtain tuning parameters		
	(3) Minimize mutual information		
ERP extraction	Minimize error in adaptive filters	Data driven	ERP components are prone to get distorted
Feature selection	(1) Filter method	Reduces overall system time complexity	Non repeatable set of features get selected
	(2) Wrapper method	Enhances accuracy	Only subject-specific selection
	(3) Embedded/Hybrid method		
Channel selection	(1) Minimum number of channels	Can lead to usage of low cost devices	Highly subjective
	(2) Region of interest-based	Enhances accuracy	Additional data reduction method required
	(3) Classification accuracy-based	Reduced system complexity	
Mode decomposition	(1) All modes sum up to form the original signal with least error	Decomposition based on frequency information	Increased time complexity
	(2) Sum of bandwidths of all modes is minimum		

As EEG is a very powerful diagnostic tool for detecting abnormal electrical discharges in the brain, its usage in the field of medicine is inevitable. Optimization has been used in various ways in such EEG-based diagnosis process and hence, this section aims at throwing light on such applications.

One of the early implementations of genetic algorithm in epileptic EEG is found in Marchesi et al. (1997). The authors utilized genetic algorithm to detect the 3 Hz spikes and slow wave complexes in the EEG. The objective function involved the following

(2) $$\begin{array}{l} f=fitness\;cases-hits \end{array}$$

where *fitness cases* corresponds to the total number of training examples and *hits* refer to the count of the matches. The stopping is thus when the count of the training cases equals to that of the hits or when the maximum number of generations are reached. An overall accuracy of 85% is seen with this setup.

The works in Wen and Zhang (2017) showed the usage of optimization in the frequency domain bin selection and in overall subset of feature selection in the analysis of epileptic EEG. A variant of genetic algorithm is used to first search for the optimal frequency ranges as features and then the features thus obtained are fused with non linear EEG features. The objective function thus aims at minimizing the linear discriminant analysis-based coefficients of the frequency bin summations done over an assortment of bins and traversed using certain constants called the slack variables. For the feature selection process, the objective function aims at minimizing the following,

(3) $$\begin{array}{l} minimize\left(FPR-\left(1-TPR\right)\right) \end{array}$$

where FPR is the false positive rate and TPR is the true positive rate.

The detection of epileptic seizures is attempted using grid search optimization as in Wang et al. (2019). The usage of optimization in this study was to tune the parameters of the random forest algorithm as it mainly generates a large number of hyperparameters and it is difficult to empirically arrive at the optimal values of these parameters. The targeted hyperparameters were number of decision trees, minimum sample leaf, maximum features, number of split features, and number of estimators. The objective function was to maximize the classification accuracy based on K-fold cross-validation technique. On similar grounds the work in Gomathi et al. (2020) worked toward detecting brain abnormalities arising due to brain stroke, brain tumor, birth defects, genetic mutation, and brain injuries using evolutionary gravitational neocognitron based optimization technique to obtain tuned parameters in a typical neural network classifier. Another attempt in optimizing a standard neural network classifier using genetic algorithm for detecting Alzheimer's disease is in Kim et al. (2005). This study made use of a single channel EEG and used rest and auditory odd ball stimulus for generating event related potentials. Standard EEG features were derived and the objective function confined to the NN architecture is used,

(4) $$\begin{array}{l} f=\frac{1}{N\times m}\overset{N}{\underset{i=1}{\sum }}\;\overset{m}{\underset{j=1}{\sum }}{\left(NO_{ij}-DO_{ij}\right)}^{2} \end{array}$$

where *NO* is the network output and *DO* is the desired output. *N* is the number of training patterns and *m* is the number of output nodes of the network. The work in Singh et al. (2019) showed the optimization of parameters in an ensemble of classifier algorithms for the sake of classifying epileptic EEG. Thus, optimization has crucial role to play in the field of medical EEG analysis.

## 5. Conclusions

This paper summarizes the various optimization approaches in BCI pipelines. It is to be noted that evolutionary optimization techniques have been widely used in the domain of EEG signal analysis. The widely used evolutionary algorithms were GA, ABC, DE, PSO, and so on. It is to be noted that these algorithms were further enhanced so as to adapt to the use-cases in BCI. The usage of evolutionary algorithms for optimizing the parameters in BCI exceeds that of linear programming-based conventional tools of optimization. The reason being that the latter assumes the starting point of the search to be well-defined, whereas in case of evolutionary schemes, the starting point is selected heuristically.

Most of the existing literature on using optimization in BCI focuses mainly on optimal feature or channel selection, and a very few works dealing with EEG preprocessing or ERP detection using optimization are found. The review aims at providing the researches in the field to have a clear understanding of the techniques of optimization applied in BCI domain so far. As a guideline for using optimization in BCI, we observe that,

- Many optimization tools are readily available which can be either used directly for BCI uses-cases or needs to be enhanced so as to obtain better outcomes. The modification or enhancement of existing optimization tools requires a lot of expertise and skill in the field and should not be altered arbitrarily which could end up providing feasible solutions to a limited set of inputs.
- The nature of task and the area of using optimization techniques should be well-studied by using the existing literature. The tables summarizing the techniques and the application area can be used in this regard.
- The optimization problem should be designed carefully so as to match closely with the domain knowledge. In most of the cases, multiobjective optimization method is required and the confusion with pareto optimal solutions should be taken care of, effectively.
- Mode decomposition of EEG signals should be done using high end machines owing to the computational demands of the algorithms. In the absence of such systems, only small portions of EEG with fewer channels can be decomposed into fewer IMFs. The number of IMFs required should be judicious and the center frequencies of each of them should be assessed to avoid unwanted realizations of the signals.

The aim of this review is to help the researchers in knowing the state of existing attempts made in optimizing the BCI pipelines. We further encourage the readers to use the references for each of the pipelines for understanding the methodologies in detail.

## Author Contributions

All authors listed have made a substantial, direct and intellectual contribution to the work, and approved it for publication.

## Conflict of Interest

The authors declare that the research was conducted in the absence of any commercial or financial relationships that could be construed as a potential conflict of interest.
